# mTOR‐mediated calcium transients affect cardiac function in *ex vivo* ischemia–reperfusion injury

**DOI:** 10.14814/phy2.14807

**Published:** 2021-03-26

**Authors:** Briana K. Shimada, Naaiko Yorichika, Jason K. Higa, Yuichi Baba, Motoi Kobayashi, Toshinori Aoyagi, Tomohiro Suhara, Takashi Matsui

**Affiliations:** ^1^ Department of Anatomy, Biochemistry, and Physiology Center for Cardiovascular Research John A. Burns School of Medicine University of Hawai‘i at Manoa Honolulu Hawai‘i USA; ^2^ Department of Cardiology and Geriatrics Kochi Medical School Kochi University Kochi Japan; ^3^ Department of Anesthesiology Keio University School of Medicine Tokyo Japan

**Keywords:** calcium, cardiomyocyte, ischemia–reperfusion, mTOR

## Abstract

The mechanistic target of rapamycin (mTOR) is a key mediator of energy metabolism, cell growth, and survival. While previous studies using transgenic mice with cardiac‐specific overexpression of mTOR (mTOR‐Tg) demonstrated the protective effects of cardiac mTOR against ischemia–reperfusion (I/R) injury in both *ex vivo* and *in vivo* models, the mechanisms underlying the role of cardiac mTOR in cardiac function following I/R injury are not well‐understood. Torin1, a pharmacological inhibitor of mTOR complex (mTORC) 1 and mTORC2, significantly decreased functional recovery of LV developed pressure in *ex vivo* I/R models (*p* < 0.05). To confirm the role of mTOR complexes in I/R injury, we generated cardiac‐specific mTOR‐knockout (CKO) mice. In contrast to the effects of Torin1, CKO hearts recovered better after I/R injury than control hearts (*p* < 0.05). Interestingly, the CKO hearts had exhibited irregular contractions during the reperfusion phase. Calcium is a major factor in Excitation‐Contraction (EC) coupling via Sarcoplasmic Reticulum (SR) calcium release. Calcium is also key in opening the mitochondrial permeability transition pore (mPTP) and cell death following I/R injury. Caffeine‐induced SR calcium release in isolated CMs showed that total SR calcium content was lower in CKO than in control CMs. Western blotting showed that a significant amount of mTOR localizes to the SR/mitochondria and that GSK3‐β phosphorylation, a key factor in SR calcium mobilization, was decreased. These findings suggest that cardiac mTOR located to the SR/mitochondria plays a vital role in EC coupling and cell survival in I/R injury.

## Graphical abstract

An overview of mTOR signaling in cardiomyocyte Excitation‐Contraction coupling.

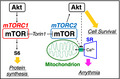

**New & noteworthy**

Previous studies showed that mTOR is a key mediator of metabolism, cell growth, and survival. However, the role of mTOR in cardiac function is not well characterized. Using cardiac‐specific mTOR‐knockout mice, we demonstrated that mTOR plays a vital role in Excitation‐Contraction coupling and cell survival following *ex vivo* ischemia–reperfusion.

## 1 INTRODUCTION

The mammalian target of rapamycin (mTOR), a member of the PI3K (phosphatidylinositol 3‐kinase)‐related protein kinase family, is a key mediator of metabolism, cell growth, and survival in a variety of organs including the heart (Laplante & Sabatini, 2012; Sciarretta et al., 2018; Suhara et al., 2017). Studies using genetic mouse models demonstrated that cardiac mTOR protects the heart against ischemia–reperfusion (I/R) injury in both *ex vivo* and *in vivo* models (Aoyagi et al., 2012, 2015). However, the role of mTOR in cardiac function in I/R injury is not characterized well.

mTOR is part of the insulin‐PI3K‐Akt signaling pathway, all of which contribute to cell survival (Sciarretta et al., 2018). mTOR forms two complexes, mTOR complex 1 (mTORC1) and mTOR complex 2 (mTORC2) (Laplante & Sabatini, 2012). These two complexes have differing roles in the cell: mTORC1 activates p70S6K/S6 and promotes protein translation and synthesis, and cellular growth (Laplante & Sabatini, 2012). mTORC2 activates Akt by phosphorylation at Ser^473^ and is known to regulate cardiomyocyte cell survival (Laplante & Sabatini, 2012). Previous studies using pharmacological manipulators of mTOR, such as rapamycin and its derivatives, have resulted in discrepancies (Sciarretta et al., 2018). Some reports demonstrated cardioprotective effects in response to mTOR inhibition (Khan et al., 2006). However, the beneficial effect of rapamycin in I/R injury is likely due to the re‐activation of PI3K/Akt signaling by disrupting the negative feedback of insulin receptor substrate 1 (IRS‐1) on the insulin receptors (Harrington et al., 2005). In fact, overexpression of Proline‐Rich AKT Substrate of 40 kDa (PRAS40), which binds to and inhibits mTORC1, protected the heart against ischemic injury, accompanied by Akt activation in the myocardium (Volkers et al., 2013). In order to clarify the role of mTOR in I/R injury, our laboratory generated cardiac‐specific transgenic mice overexpressing mTOR (mTOR‐Tg) (Aoyagi et al., 2012). Our previous publication using the mTOR‐Tg mice demonstrated that overexpression of mTOR was cardioprotective in both *ex vivo* and *in vivo* settings, signifying mTOR is *sufficient* to protect the heart against I/R injury and mitigate cell death (Aoyagi et al., 2012). However, the role of mTOR in cardiac function and physiology parameters such as calcium (Ca^2+^) transients in I/R injury is not characterized well.

In this study, we determine whether mTOR is *necessary* to preserve cardiac function against I/R injury using cardiac‐specific mTOR‐KO (CKO) mice. Surprisingly, the CKO hearts recovered better after I/R injury than control hearts; however, CKO CMs had weaker contractions, smaller Ca^2+^ transients, and less relative sarcoplasmic reticulum (SR) Ca^2+^ concentrations. Overall, our results indicate that mTOR may have a novel role in Ca^2+^ regulation.

## 2 MATERIALS AND METHODS

### 2.1 Animal models

Animal experiments in this study were approved by the Institution Animal Care and Use Committees of the University of Hawaii (Honolulu, HI). This investigation confirmed with the National Institutes of Health *Guide for the Care and Use of Laboratory Animals* (NIH Pub. No. 85‐23, Revised 1996). Breeding pairs of floxed mTOR mice (mTOR^fl^; B6.129S4‐Mtor^tm1.2Koz^/J) were obtained from Jackson Laboratories and inbred to generate mTOR^fl/fl^ mice and were further inbred for more than ten generations before use. These mice contain *LoxP* sites that flank exons 1‐5 of the mTOR gene (Gangloff et al., 2004). Since these exons contain the transcription start site, Cre‐mediated deletion of these exons results in the loss of mTOR. CM specific mTOR‐KO mice were then generated by crossing homozygous mTOR^fl/fl^ mice with homozygous transgenic mTOR^fl/fl^ mice expressing a tamoxifen‐inducible Cre recombinase fused to two mutated estrogen receptors under the transcriptional control of the α‐myosin heavy chain promoter (α‐MHCmerCremer; Tg(Myh6‐cre/Esr1)1Jmk/J). This cross‐yielded mTOR^fl/fl^ mice expressing Cre (CKO) and their littermate Cre‐ controls (Con), which did not express Cre. At 6–8 weeks of age, these mice were placed on a tamoxifen chow diet consisting of 250 mg/kg of tamoxifen that provided 40 mg tamoxifen per kg body weight per day, assuming a normal 25 g body weight and 3–4 g intake, as done previously (Andersson et al., 2010). The food was monitored every 2–3 days to ensure that it was being consumed. Mice were kept on this diet for two weeks before a normal chow diet was resumed. Any animal losing more than 20% of its body weight was immediately removed from the study.

### 2.2 Echocardiography

Echocardiography was performed on non‐anesthetized mice using a 13 L high‐frequency linear transducer (Vevo 2100 Imaging system, Visual Sonics, Toronto, ON, Canada) using a MS400 transducer (18–38 MHz) as previously described (Aoyagi et al., 2012, 2015; Song et al., 2010). After confirming wall motion of all LV area by B‐Mode, M‐mode was acquired. Left ventricular interior diameter dimension (LVIDd) and % fractional shortening (%FS) were measured for baseline analysis of cardiac structure and function for Con and CKO mice, as described previously (Lindsey et al., 2018).

### 2.3 Isolation of adult murine ventricular myocytes

Con or CKO male mice (8–12 weeks of age) were anesthetized with 2,2,2‐tribromoethanol (TBE). The heart was quickly removed from the chest and retrograde perfused at a constant flow rate of 3 ml/min at 37°C for 2–3 min with a Ca^2+^‐free bicarbonate‐based buffer containing 120 mM NaCl, 5.4 mM KCl, 1.2 mM MgSO_4_, 1.2 NaH_2_PO_4_, 5.6 mM glucose, 20 mM NaHCO_3_, 10 mM 2,3‐butanedione monoxime (BDM; Sigma) and 5 mM taurine (Sigma), gassed with 95% O_2_‐5%CO_2_ to washout remaining Ca^2+^ in the heart. Following perfusion with the Ca^2+^‐free buffer, enzymatic digestion was initiated by perfusing with a collagenase buffer containing 0.4 mg/ml collagenase type B (Roche, Basel, Switzerland), 0.3 mg/ml collagenase type D (Roche), and 0.03 mg/ml protease type XIV (MilliporeSigma) in 50 ml of Ca^2+^‐free perfusion buffer. All solutions were filtered with a 0.2 µM filter. Hearts were perfused with the collagenase buffer for 15–20 min until the heart was fully digested. Collagenase buffer was then washed out by perfusing again with Ca^2+^‐free buffer for 2–3 min. Cardiomyocytes (CMs) were isolated by mechanically teasing the cells apart. They were then gently triturated with a plastic transfer pipette and filtered using a sterile 100 µM filter (Song et al., 2010). Myocytes were then allowed to settle by gravity. CMs were then snap‐frozen in liquid nitrogen for later use in Western blot analysis.

### 2.4 Ex vivo I/R in Langendorff‐perfused hearts

Male Con and CKO mice ages 12–20 weeks old were heparinized with 1000 IU/kg and anesthetized with 250 mg/kg of TBE diluted in sterile PBS. Hearts were excised and subjected to an *ex vivo* Langendorff perfusion model as previously described (Aoyagi et al., 2012). After retrograde perfusion was established at a constant pressure (80 mmHg), hearts were perfused with a modified Krebs–Henseleit buffer (11 mM glucose, 118 mM NaCl, 4.7 mM KCl, 2.0 mM CaCl_2_, 1.2 mM MgSO_4_, 1.2 mM KH_2_PO_4_, 25 mM NaHCO_3_, and 0.5 mM EDTA) equilibrated with 95% O_2_‐5%CO_2_ at 37°C to yield a pH of 7.4. A water‐filled balloon catheter was introduced into the left ventricle (LV) to record LV pressure (PowerLab, AD Instruments, Denver, CO). The volume of the coronary sinus effluent in the collected perfusate was measured to determine the coronary flow rate. For the *ex vivo* I/R model, hearts were perfused for 15 min, and the flow was eliminated for 20 min, followed by reperfusion for 40 min. To calculate the percent left ventricular developed pressure recovery (%LVDP), the LVDP was compared at baseline and at 10, 20, 30, and 40 min of reperfusion as previously reported (Aoyagi et al., 2012). In some groups, hearts were perfused for 10 min and treated with 100 nM Torin1 (Cayman Chemical, Ann Arbor MI) for 20 min. The flow was eliminated for 20 min followed by 40 min of reperfusion. To calculate the average variance of contraction, the variance was measured from four separate 10‐min intervals. The average LVDP $\left(\overset{‐}{x}\right)$ over the entire 10‐min interval was calculated. Then the change from the average LVDP was calculated for each individual peak $\left(x_{i}\right)$. n indicates the total number of peaks across the 10‐min interval. These values were then inputted into the following formula used to calculate the variance: $S^{2}=\frac{\Sigma {\left(x_{i}-\bar{x}\right)}^{2}}{\left(n-1\right)}$. The variance for each sample was then averaged to get the average variance for each group.

### 2.5 Assay for the level of creatine kinase (CK) in ex vivo perfused hearts in I/R injury

The enzyme activity of CK was determined in the effluent collected at baseline and 40 min of reperfusion by enzyme activity kits as previously done (CK; BioAssay Systems, Hayward, CA) (Aoyagi et al., 2012, 2015).

### 2.6 Subcellular fractionation of whole heart tissue

Hearts were isolated from Con or CKO mice 12–20 weeks of age and placed in fiber relaxation buffer (100 mM KCl, 5 mM EGTA, 5 mM HEPES/KOH, pH = 7.5) for 10 min. Hearts were dried and homogenized with a glass dunce homogenizer in SPHEM‐A buffer (250 mM sucrose, 20 mM HEPES/KOH, pH = 7.5; 10 mM KCl, 1.5 mM MgCl, 1.5 mM Na EGTA, 2.5 mM Na EDTA, 1 mM DTT, 0.1 mM PMSF). The homogenate was centrifuged at 750 *g* for 10 min at 4°C. The resulting supernatant was collected and pellets were washed with sucrose‐free buffer (SPHEM‐A buffer without sucrose). These pellets were saved and labeled as the “nuclear fraction.” The supernatant was then centrifuged at 12,500 *g* for 15 min at 4°C. Again, the resulting supernatant was collected and the pellet was washed and resuspended with 1 ml SPHEM‐A buffer. This fraction was centrifuged at 4000 *g* for 15 min at 4°C. The supernatant from this fraction was discarded and the pellet was saved as the “mitochondria (mito) heavy fraction.” The supernatant that was collected in the previous step was then centrifuged at 100,000 *g*. The resulting supernatant was collected and labeled as the “cytosolic fraction,” whereasthe pellet was labeled as the “SR/mito fraction” (Desai et al., 2002).

### 2.7 Western blot analysis

Whole hearts or isolated cardiomyocytes were harvested, snap frozen, and crushed in liquid nitrogen. Tissue was homogenized in ice‐cold lysis buffer (Cell Signaling, Danvers, MA) as previously described (Aoyagi et al., 2012, 2015; Song et al., 2010). Protein concentrations were measure using the Bradford method (BioRad, Hercules, CA). Subcellular fractions were obtained as described above. SDS‐PAGE was performed under reducing conditions on 4–20% gradient gels (Bio‐Rad). Proteins were then transferred to a PVDF transfer membrane with fluorescent capability (MilliporeSigma, St. Louis, MO). Blots were blocked with 5% fish gelatin for 1 hour at room temperature prior to antibody incubation. Blots were then incubated with primary antibodies 18–20 h at 4°C. Blots were then incubated with either a green fluorescent donkey anti‐mouse secondary antibody or a red fluorescent goat anti‐rabbit secondary antibody. Signal was detected using a fluorescent reader and Image Studio by LI‐COR (Lincoln, NE). The primary antibodies were used for immunoblot analysis at the following specified dilutions: mTOR (Cell Signaling) at 1:1000, phospho‐S6 (pSer235/236, Cell Signaling) at 1:1000, total S6 (Cell Signaling) at 1:1000, phospho‐Akt (pSer473, Cell Signaling) at 1:1000, Akt (Cell Signaling) at 1:1000, Gapdh (Santa Cruz, Dallas, TX) at 1:1000, VDAC (Cell Signaling) at 1:1000, phospho‐GSK‐3β (pSer9, Cell Signaling) at 1:1000, GSK‐3 (Cell Signaling) at 1:1000, SERCA2a (Santa Cruz) at 1:1000, phospho‐PLN (phospholamban, Cell Signaling) at 1:1000, PLN (Cell Signaling) at 1:1000, Cre (MilliporeSigma) at 1:10,000, phospho‐RYR2 (pSer2808 ryanodine receptor 2, Badrilla, Leeds, UK) at 1:2000, and RYR2 (Thermo Scientific, Waltham, MA) at 1:1000.

### 2.8 Measurement of Ca^2+^ transients and sarcomere length shortening

Adult cardiomyocytes were isolated from either Con or CKO mice, as done previously (Baba et al., 2018). Myocytes were loaded with the Ca^2+^ indicator Fura 2‐AM (5 µM) for 15 min at 25°C. Myocytes attached to a coverslip were placed in a chamber mounted on the stage of an inverted microscope (Nikon Eclipse, Melville, NY) and perfused at approximately 2 ml/min at 37°C with a buffer containing 10 mM glucose, 137 mM NaCl, 5.4 mM KCl, 0.5 mM MgCl_2_, 10 mM HEPES (pH = 7.4), and 1.2 mM CaCl_2_. The cells were field‐stimulated at a frequency of 2 Hz. A video‐based edge detector was used to capture and convert changes in cell length during shortening and re‐lengthening into an analog voltage signal (IonOptix, Milton, MA). Cell contractions were assessed using the following indicators: peak shortening, time to 90% PS (TPS), time to 90% re‐lengthening (TR90), and maximal velocities or shortening and re‐lengthening (±dL/dt). Ca^2+^ was evaluated by examining the Fura‐2 emission ratio of intracellular to extracellular Ca^2+^ as previously described (Shi et al., 2010).

### 2.9 Caffeine stimulation of isolated adult murine ventricular CMs

CMs were field stimulated at 0.5 Hz to steady state for 1 min before pacing was stopped for 30 s and 10 mmol/L caffeine was immediately applied. The caffeine was allowed to washout for another 30 s and the CMs were paced again at 0.5 Hz to re‐attain the same steady state (Zhang, Guo, et al., 2010).

### 2.10 Statistical analysis

Results were analyzed using Graph Pad's PRISM software. Statistical tests were applied according to experimental design, as indicated in the figure legends. Comparisons between two groups were analyzed by Student's *t* test. For comparison of multiple groups, one‐way ANOVA was used. Tukey's post hoc test was used as a post‐test for one‐way ANOVA. *P* values are also shown in the figures or graphs. All results are reported as means ± SEM.

## 3 RESULTS

### 3.1 Torin1 treatment decreases the %LVDP recovery of wild‐type mice and reduces cell death

**FIGURE 1:**
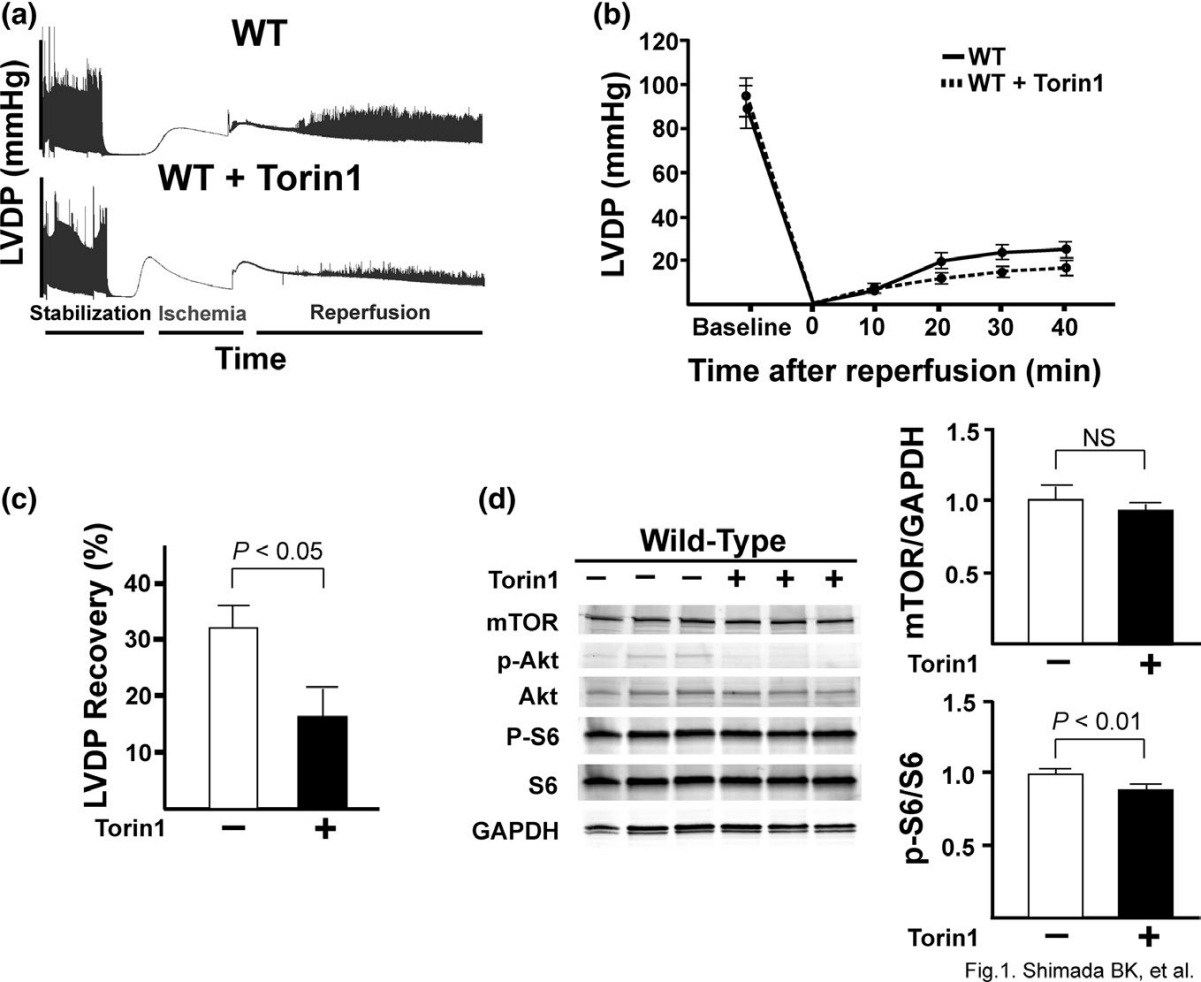
Torin1 significantly decreases %LVDP of wild‐type mice and decreases downstream signaling of mTORC1 and mTORC2. Wild‐type C57BL/6J mice were subjected to *ex vivo* Langendorff and treated with or without 100 nM Torin1. (a) Representative tracings from the Torin1 experiments. (b) Change in left ventricular developed pressure at baseline and throughout all 40 min of reperfusion. (c) Quantification of the %LVDP recovery of wild‐type mice treated with or without Torin1. The LVDP at baseline and reperfusion at 40 min of reperfusion were compared to calculate the %LVDP. N = 7 (WT) and 8 (WT + Torin1). *p* < 0.05 by Student's *t* test. (d) Immunoblot showing signal transduction and phosphorylation of downstream targets (p‐Akt and p‐S6) of mTORC1 and mTORC2 in hearts treated with Torin1. Bar graphs represent the mean densitometric analysis of mTOR and p‐S6. mTOR was normalized to GAPDH. p‐S6 was normalized to total S6. *P*‐values are displayed on graphs as determined by Student's *t* test. N = 3 for all groups.

Rapamycin, an mTORC1 inhibitor, protects the heart against *ex vivo* I/R injury (Das et al., 2012; Khan et al., 2006). Torin1 is a highly potent and selective mTOR inhibitor that targets and inhibits both mTORC1 and mTORC2 (Liu et al., 2010; Volkers et al., 2013). To define the role of both mTOR complexes, we tested the effects of Torin1 in the settings of an *ex vivo* acute MI model. We subjected C57BL/6J (wild‐type) hearts to *ex vivo* Langendorff I/R injury and pre‐treated the hearts with or without Torin1 for 20 min before I/R (Figure 1a,b). Torin1 significantly decreased the percent LV developed pressure (LVDP) recovery (maximum LV developed pressure recovery: 31.3 ± 3.9% vs. 16.6 ± 4.0% for control vs. Torin1, *p* < 0.05; Figure 1c), suggesting that acute inhibition of both mTOR complexes was detrimental to the heart in I/R injury. To verify the pharmacological effects of Torin1, we assessed the mTOR signaling pathway in Torin1‐treated hearts. Western blotting demonstrated that treatment with Torin1 completely inhibited the phosphorylation of Akt, while it slightly but significantly decreased the level of phospho‐S6 (p‐S6) (Figure 1d). The significant decrease in S6 and Akt phosphorylation demonstrated that Torin1 treatment was effective at inhibiting downstream mTORC1/2 signaling, predominantly in the mTORC2 pathway. Taken together, these results show that acute inhibition of mTOR suppresses functional recovery of *ex vivo*‐perfused wild‐type hearts.

### 3.2 Characterization of cardiac‐specific mTOR knockout mice

**FIGURE 2:**
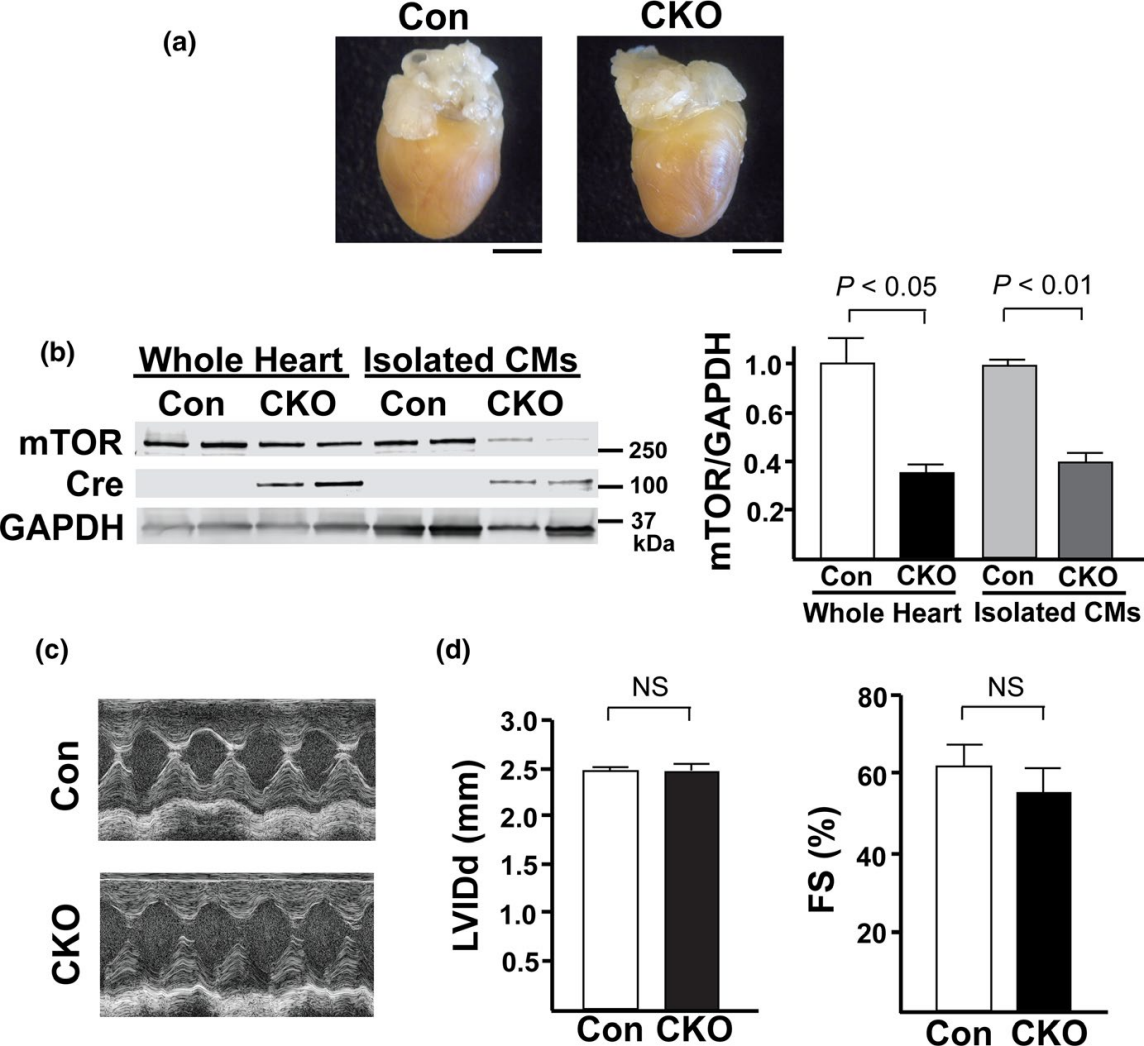
Characterization of CKO mice. (a) Representative pictures of whole hearts isolated from Con and CKO mice. Scale bars =2 mm. (b) *Left*. Representative immunoblot showing a small decrease in mTOR expression in whole hearts and a significant decrease in the expression of mTOR in isolated CMs and in the whole heart compared to control. *Right*. Densitometric quantification of the amount of mTOR in whole heart and isolated CMs. N = 6 in each group. (c) Representative M‐mode traces from baseline echocardiography analysis of control and CKO mice. CKO mice had normal contractions that were similar to controls. (d) *Left*. CKO mice LVIDd was comparable to controls. *Right*. %FS was similar between control and CKO mice. N = 7 in each group.

Our data using Torin1 suggested that mTOR was necessary to protect the heart against I/R injury. To verify this in a genetic model, we created a tamoxifen‐inducible, cardiac specific, mTOR knockout mouse (CKO). In order to confirm the gene silencing of mTOR in our CKO mice, we examined both the whole hearts and isolated CMs from CKO mice and compared them with control (Con) mice that lacked the Cre enzyme (Cre‐). Western blotting demonstrated a significant reduction of mTOR expression in both whole hearts and isolated CMs in our CKO mice (*p* < 0.05 for whole heart CKO vs. Con and *p* < 0.01 for isolated CMs; Figure 2b). Residual mTOR expression observed is likely a consequence of cells that did not have a recombined mTOR allele or from non‐cardiac cells such as endothelial cells or fibroblasts. Littermate mice were then analyzed at 12–14 weeks for baseline cardiac function and structure (Tables 1 and 2). Examination of whole hearts from CKO and Con mice revealed no gross abnormalities in CKO hearts as they were similar in size and shape as Con hearts (Figure 2a). Heart weight to tibia length ratio (HW:TB) was also similar between CKO and Con mice indicating the hearts between the two groups were relatively the same size (Table 1). Likewise, there was also no difference in cardiac function, either. Echocardiography and subsequent analysis demonstrated no significant differences in various measures of structure and function (Table 2, Figure 2c,d). Cardiac function was evaluated as percent fractional shortening (%FS: 61.1 ± 2.3 vs. 53.4 ± 2.9% for Con vs. CKO mice; Figure 2d) and left ventricular interior diastolic dimension (LVIDd: 3.03 ± 0.03 vs. 3.02 ± 0.07 mm; Figure 2d). These measurements of cardiac function were similar between CKO and Con hearts. Therefore, genetic deletion of cardiac mTOR did not result in any structural abnormalities nor modify cardiac function at baseline.

**TABLE 1:** General measurements

	Con (N = 24)	CKO (N = 24)
Body Weight (g)	26.93 ± 0.39	26.18 ± 0.42
Heart Weight (mg)	19.8 ± 0.6	19.33 ± 0.6
HW:TB (mg/mm)	8.83 ± 0.32	8.45 ± 0.24

Abbreviations: HW:TB, heart weight:tibia length.

**TABLE 2:** Baseline echocardiograph parameters

	Con (N = 7)	CKO (N = 7)
LVIDd (mm)	3.03 ± 0.028	3.02 ± 0.069
LVIDs (mm)	1.19 ± 0.078	1.39 ± 0.97
%FS (%)	61.13% ± 2.28	53.39% ± 2.90
HR (bpm)	710.71 ± 10.48	710.62 ± 4.57

Abbreviations: LVIDd, LV internal dimension diastole; LVIDs, LV internal dimension systole; %FS, % fractional shortening; HR, heart rate.

### 3.3 Loss of mTOR leads to better recovery in ex vivo Langendorff I/R model

**FIGURE 3:**
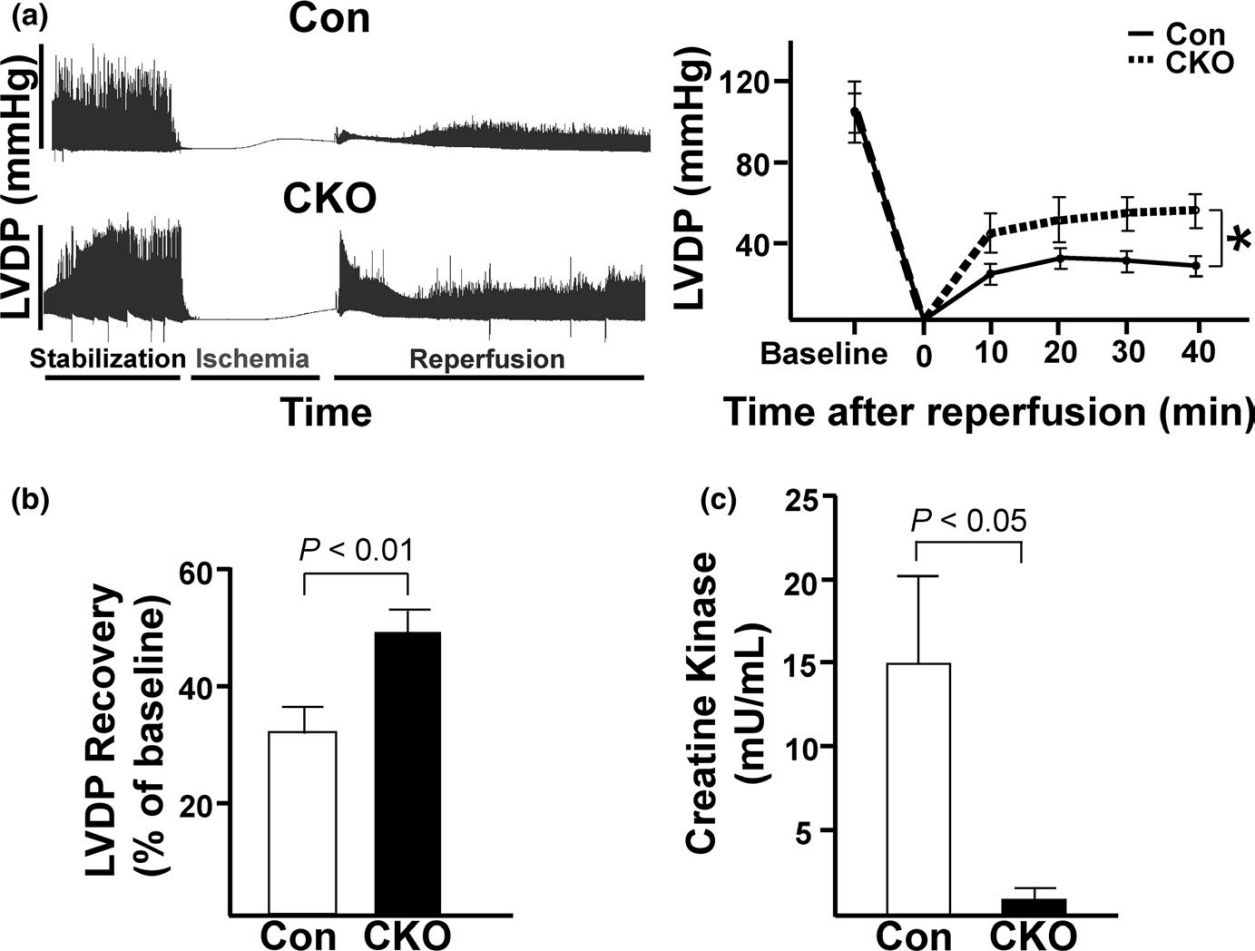
CKO mice have better %LVDP recovery after *ex vivo* I/R. (a) *Left*. Representative tracing showing LVDP throughout the Langendorff experiment. *Right*. Quantification of LVDP at baseline and after every 10 min of reperfusion. (b) Quantification of the %LVDP recovery for control vs. CKO at 40 min of reperfusion. (c) Activity of creatine kinase (CK) in the effluent collected during the reperfusion period. To compare enzyme activities immediately after *ex vivo* I/R injury between Con and CKO hearts, effluents from either Con or CKO hearts were collected after 40‐min reperfusion. *p < 0.05. All other p‐values displayed on graphs. N = 7 for each group.

Since CKO hearts did not exhibit any changes in cardiac function or structure at baseline, we next wanted to challenge our CKO hearts with a model of I/R injury. We used *ex vivo* Langendorff to simulate and measure cardiac function in CKO hearts. There was no significant difference in baseline function between Con and CKO hearts before the induction of I/R (Table 3). After 20 min of global ischemia, followed by 40 min of reperfusion, surprisingly, the CKO hearts recovered significantly better than Con hearts (Figure 3a). At 40 min of reperfusion, percent %LVDP recovery was significantly greater in CKO hearts (%LVDP 33.3 ± 4.8 vs. 51.5 ± 3.1%, *p* < 0.01; Figure 3b). Creatine kinase (CK), a myocardial injury marker, was also significantly reduced in the CKO hearts, confirming that CKO hearts had less CM cell death than Con hearts (14.8 ± 6.1 vs. 0.9 ± 0.7 mU/ml, *p* < 0.05; Figure 3c).

**TABLE 3:** Baseline *ex vivo* Langendorff parameters

	Con (N = 7)	CKO (N = 7)
Dp/Dt (max)	5824.36 ± 1040.97	5288.40 ± 630.57
Dp/Dt (min)	−3544.57 ± 242.41	−3238.95 ± 347.38
LVSP (base) mmHg	113.04 ± 13.56	113.07 ± 8.93
LVEDP (base) mmHg mmHg	6.45 ± 1.01	5.81 ± 1.15
LVDP (base) mmHg	106.67 ± 13.95	107.26 ± 9.83
Heart Rate (bpm)	369.83 ± 31.92	343.45 ± 18.32
Coronary Flow (ml/min)	4.30 ± 0.967	6.41 ± 0.856

Abbreviations: Dp/Dt (max), maximum derivative of change in systolic pressure over time; Dp/Dt (min), minimum derivative of change in diastolic pressure over time; LVSP, left ventricular systolic pressure; LVEDP, left ventricular end‐diastolic pressure; LVDP, left ventricular developed pressure.

### 3.4 mTOR CKO hearts have irregular contractility following I/R

**FIGURE 4:**
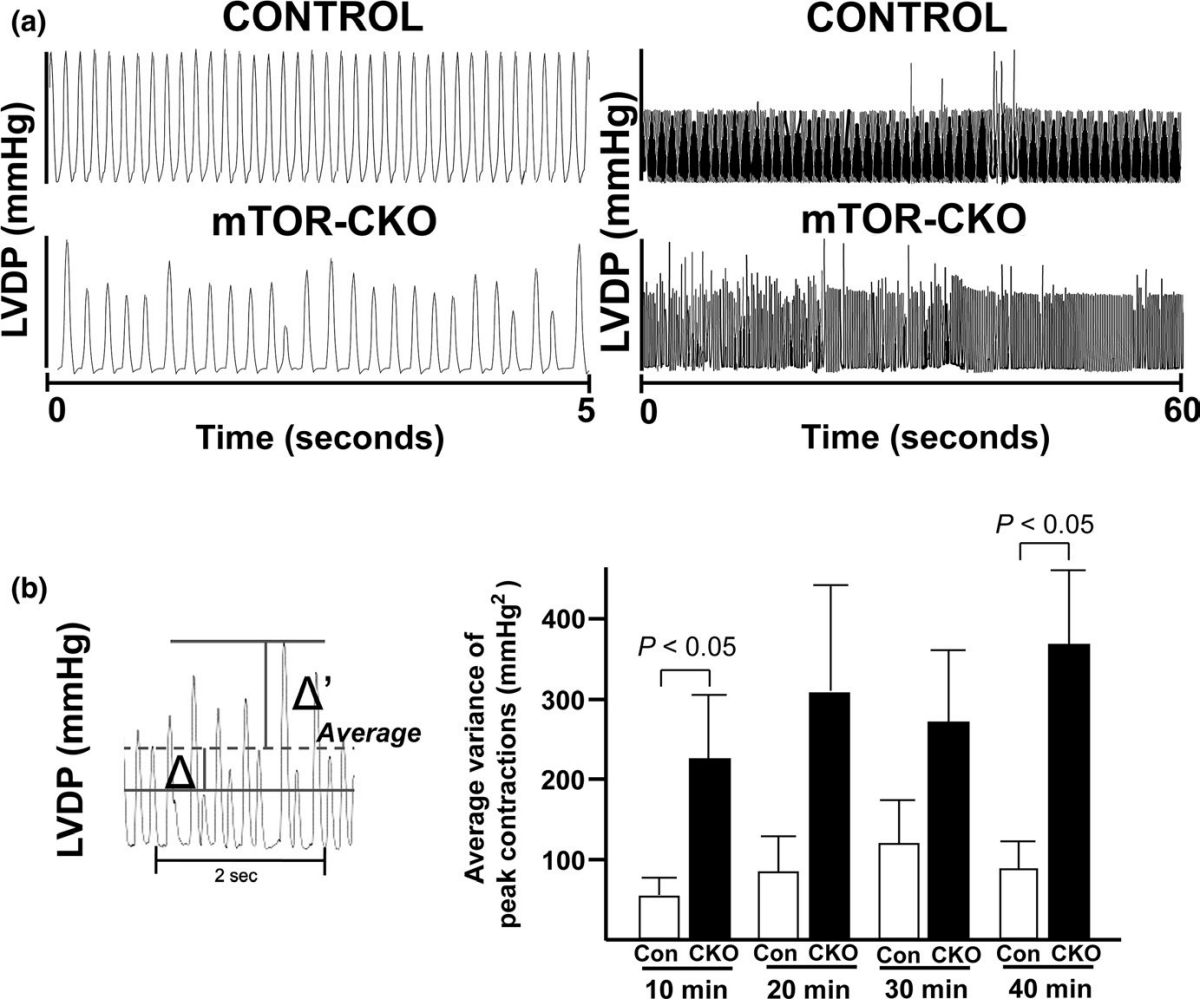
CKO hearts have irregular contractility following I/R. (a) *Left*. Representative tracing showing the difference in peak sizes between the control and CKO hearts taken over a 5‐s period. *Right*. Representative tracing showing the increased variance of contractions CKO hearts have after I/R injury. Representative tracings were taken over a 1‐min period during the last 10 min of reperfusion. (b) *Left*. Representative diagram displaying how to calculate the variance. Dashed line indicates the average LVDP across the entire interval. Δ and Δ′ indicate the change from the average LVDP for each individual peak. The average variance of peak contractions was then calculated with the formula described in *Materials and Methods*. *Right*. Quantification of the average variance of contractions. N = 7 for each group. P‐values listed on graph as determined by Student's *t* test.

Despite the CKO hearts having better post‐I/R recovery than Con hearts, we also noticed that intriguingly, the CKO hearts appeared to have irregular contractility following I/R (Figure 4a). We quantified this irregularity using the formula described in Materials and Methods, and found that CKO hearts had significantly increased variance of contraction pressure during the first ten and the last ten minutes of reperfusion (52.7 ± 19.3 vs. 223.8 ± 66.5 and 76.7 ± 32.1 vs. 348.1 ± 99.9 mmHg^2^, *p* < 0.05; Figure 4b). Previous studies using isolated rat hearts have shown that reperfusion‐induced arrhythmias are associated with alterations in intracellular Ca^2+^ regulation (Said et al., 2011). Additionally, previous studies show that mTOR has an indirect role in Ca^2+^ regulation (Betz et al., 2013; MacMillan & McCarron, 2009). Based on those reports and our findings, we hypothesized that mTOR might have a role in regulating Ca^2+^ transients.

### 3.5 Cardiomyocytes isolated from mTOR‐KO mice have weaker contractions and smaller Ca^2+^ transients

**FIGURE 5:**
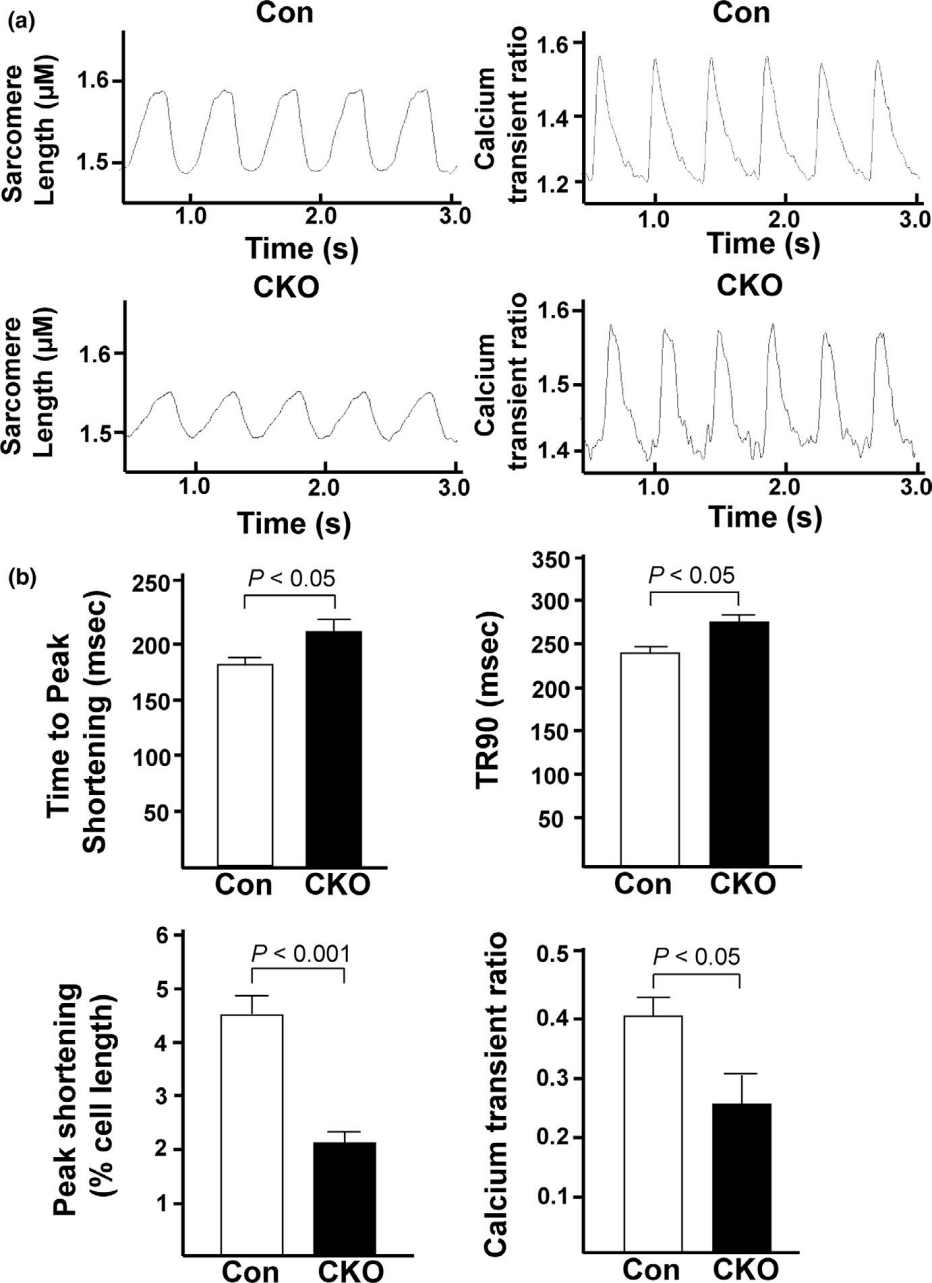
Cardiomyocytes isolated from CKO mice exhibit weaker contractions and smaller calcium transients. (a) Representative traces of sarcomere length shortening and Ca^2+^ transients in CMs isolated from Con or CKO mice. (b) Quantification of the sarcomere length and calcium transient tracings. The following parameters were quantified for each group: time to peak shortening, time to 90% relaxation, peak shortening, and calcium transient ratio. N = 3 independent experiments, 8–12 cells total for both groups. *P*‐values are all displayed on graphs.

Next, we determined the effect of mTOR knockout on CM contraction and Ca^2+^ transients. To do this, we isolated CMs from Con and CKO hearts and loaded them with Fura2‐AM, a Ca^2+^ sensitive dye, before measuring contractility and Ca^2+^ transients using the IonOptix system (Figure 5a). We found that CKO CMs had significantly weaker contractions as indicated by percent peak shortening (4.6 ± 0.4 vs. 2.1 ± 0.2%, *p* < 0.001 for Con vs. CKO CMs; Figure 5b), time to peak shortening (181.2 ± 5.4 vs. 205.3 ± 0.2 ms, *p* < 0.05), and time to 90% relaxation (242.1 ± 6.8 vs. 268.4 ± 7.5 ms, *p* < 0.05). The Ca^2+^ transient ratio was also significantly smaller in CKO CMs (0.41 ± 0.04 vs. 0.26 ± 0.06; *p* < 0.05 for Con vs. CKO CMs). Together, these data indicate that mTOR is necessary to maintain normal contractions and Ca^2+^ transients.

### 3.6 mTOR knockout cardiomyocytes have lower relative SR Ca^2+^ content

**FIGURE 6:**
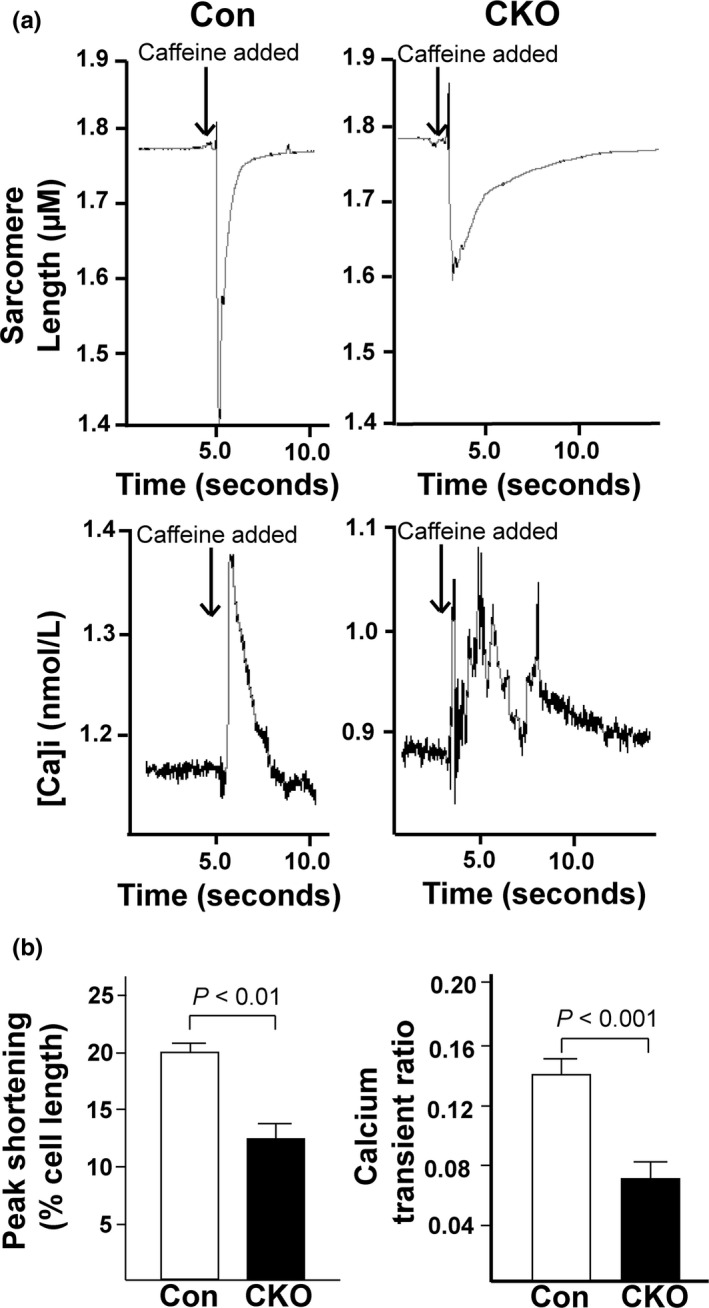
CKO CMs have lower relative SR calcium content. (a) Representative images from caffeine experiments. *Upper*. Representative tracing of sarcomere length from Con and CKO CMs stimulated with caffeine. *Lower*. Representative tracing of Ca^2+^ transients from Con and CKO CMs stimulated with caffeine. (b) Quantification of the peaks resulting from caffeine stimulation. The following parameters were quantified for each group: peak shortening and calcium transient ratio. N = 12 cells analyzed from 3 (Con) and 4 (CKO) mice. P‐values are displayed on graphs as determined by Student's *t* test.

Based on our *ex vivo* Langendorff and IonOptix data, we hypothesized that the better recovery and smaller Ca^2+^ transients of the CKO hearts might be due to a decreased amount of Ca^2+^ in the SR. In I/R injury, Ca^2+^ is one of the major triggers to open the mitochondrial permeability transition pore (mPTP) and induce programmed necrosis (Morciano et al., 2015). Lower baseline SR Ca^2+^ concentrations would result in a smaller amount of Ca^2+^ entering into the mitochondria in response to I/R and therefore, less cell death would occur in the myocardium. This dampening of mitochondrial Ca^2+^ influx and protection against mPTP formation could explain the better recovery of the CKO hearts in the *ex vivo* I/R injury model, and the lower amount of creatine kinase after I/R injury. To determine relative SR Ca^2+^ content, we stimulated Con and CKO CMs with caffeine, a Ca^2+^‐induced Ca^2+^ release stimulator (Figure 6a). We found CKO CMs had significantly lower SR Ca^2+^ (0.15 ± 0.01 vs. 0.07 ± 0.01 for Con vs. CKO CMs, *p* < 0.001, Figure 6b). Peak shortening was also significantly reduced in CKO CMs, indicating they did not completely contract when stimulated with caffeine, as compared to Con CMs (20.0 ± 1.6 vs. 13.3 ± 1.9% for Con vs. CKO CMs, *p* < 0.05, Figure 6b). Together, these data demonstrate CKO CMs have significantly less SR Ca^2+^ content, which could potentially explain the better recovery of the CKO hearts in *ex vivo* I/R, but at the cost of less efficient and more irregular contractions at the cellular level.

### 3.7 Excitation‐Contraction (EC)‐coupling proteins are unchanged in CKO hearts

**FIGURE 7:**
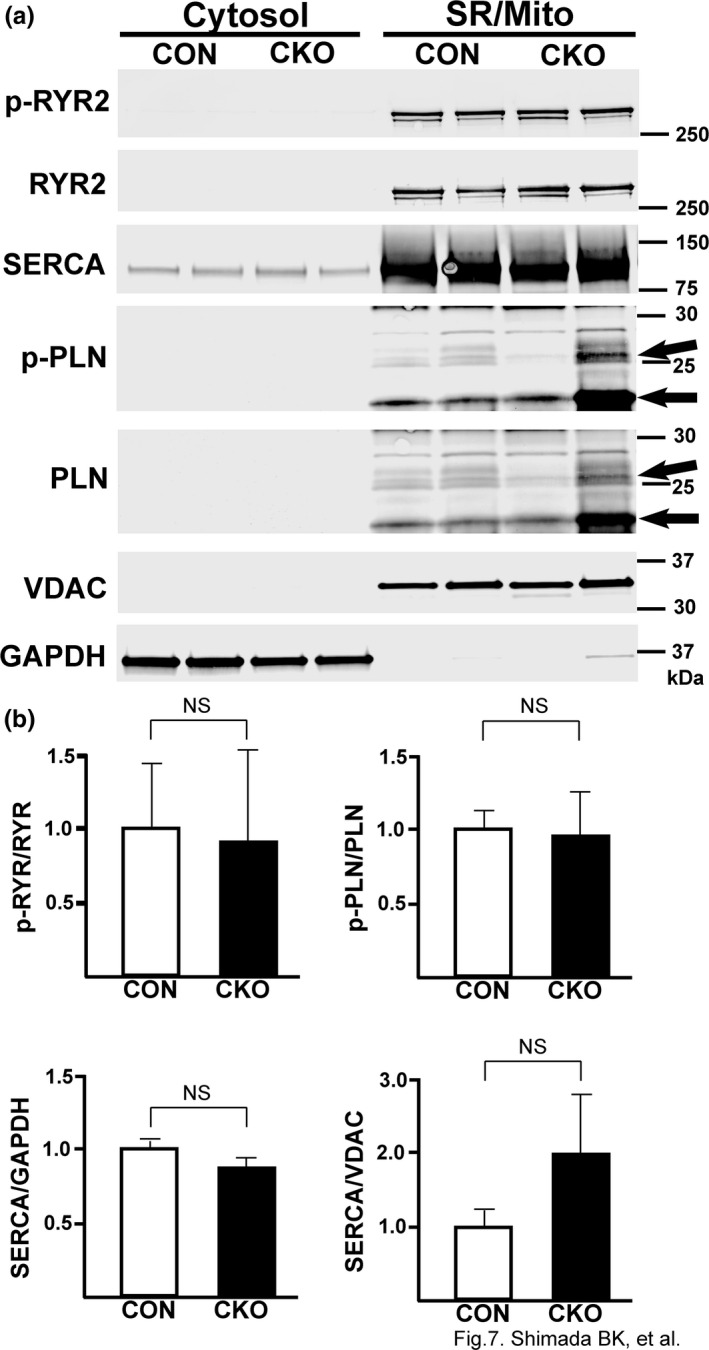
EC Coupling proteins are unchanged in CKO hearts. (a) Representative immunoblot from control and CKO hearts that underwent ultracentrifugation to obtain subcellular fractions. VDAC was used as a loading control for the SR/mito fraction while GAPDH was used as the loading control for the cytosolic fraction. (b) Quantification of the amount of p‐RYR, p‐PLN, and SERCA in controls and CKO hearts. N = 6 for each group. *P*‐values are shown on graphs.

Based on our findings that CKO CMs have smaller Ca^2+^ transients and lower relative SR Ca^2+^, we hypothesized that the localization or expression of one or more proteins involved in EC‐coupling might be altered in CKO CMs. Therefore, we used a subcellular fractionation protocol to determine the amounts of various EC‐coupling proteins in the SR and mitochondrial subcellular fractions. We found that CKO CMs had no significant changes in SERCA (sarcoendoplasmic reticulum calcium transport ATPase), p‐PLN (phospholamban) or p‐RYR (ryanodine receptor) (Figure 7a,b). As a result, we concluded from this data that alterations in EC‐coupling protein expression were not responsible for altered Ca^2+^ handling in CKO CMs, and another mechanism was responsible for the decreased amount of Ca^2+^ in the SR of the CKO CMs.

### 3.8 GSK‐3β is activated in CKO CMs at mitochondrial‐associated membranes

**FIGURE 8:**
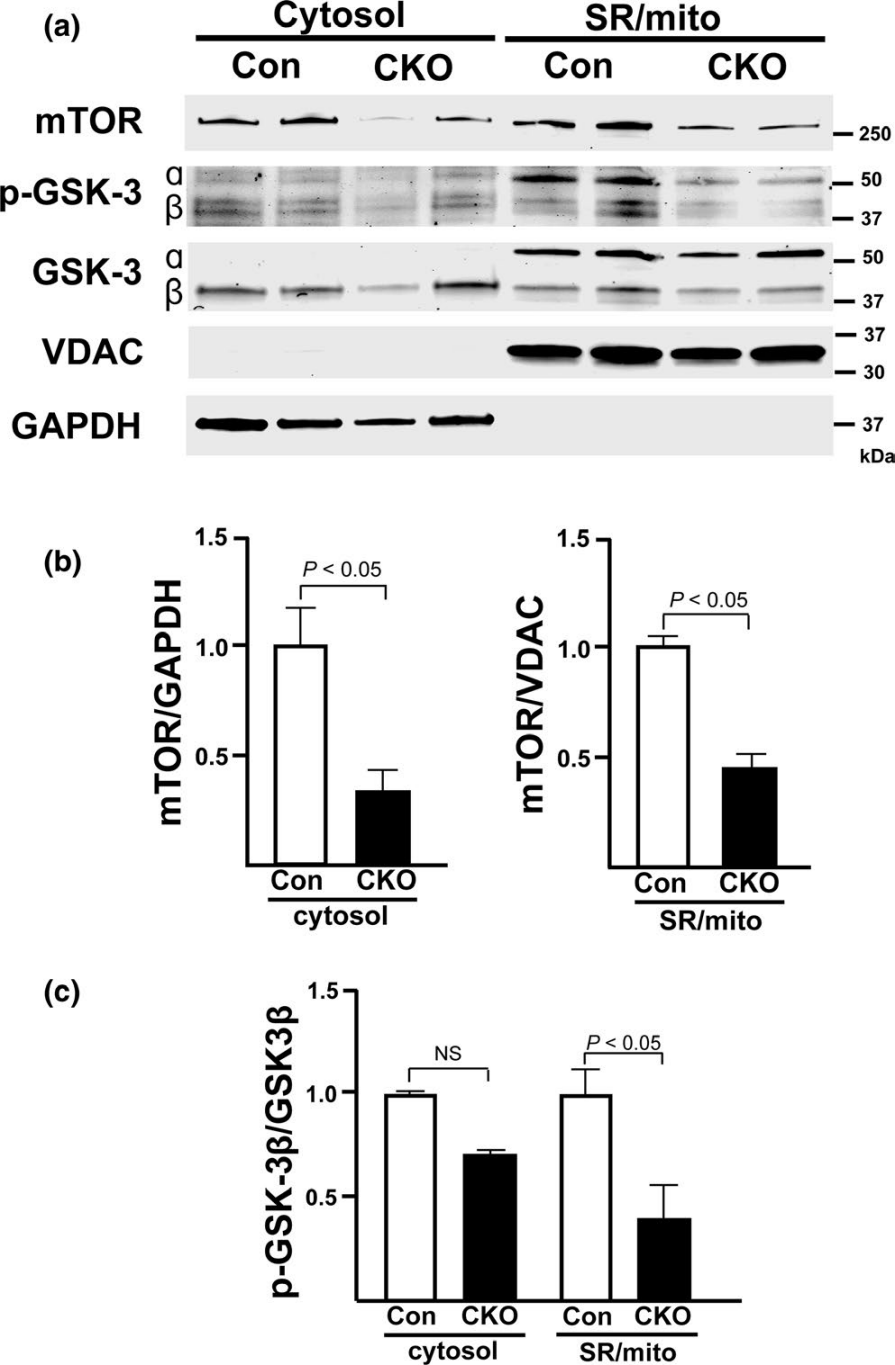
mTOR localizes to the SR/mitochondria (mito) and p‐GSK‐3β is activated in CKO CMs at mitochondrial associated membranes. (a) Representative immunoblot confirming mTOR localizes to both the SR/mito and the cytosol. (b) Densitometric quantification of the amount of mTOR in the cytosol and SR/mito fractions. mTOR was normalized to GAPDH in the cytosol fraction or VDAC in the SR/mito fraction. (c) Densitometric quantification of the amount of p‐GSK‐3β in the cytosolic and SR/mito fractions. p‐GSK‐3β was normalized to total GSK‐3β. N = 6 (Con and CKO cytosolic fractions) and 5 (Con and CKO SR/mito fractions). *P*‐values are presented on graphs.

As none of the EC‐coupling proteins evaluated were unchanged in CKO hearts, we hypothesized that another signaling pathway was affected by the loss of mTOR. One report suggested that pharmacological inhibition of GSK‐3β reduced the interaction of Inositol trisphosphate receptor (IP3R) with other partners of the SR/mitochondria (SR/mito) complex and may limit cytosolic and mitochondrial Ca^2+^ overload (Gomez et al., 2016). Therefore, we used a subcellular fractionation protocol to determine the amount of phospho‐GSK‐3β (p‐GSK‐3β) located at the SR/mito interface and to confirm knockout of mTOR at the protein level (Figure 8a). We found that CKO hearts had less mTOR in both the cytosolic and SR/mito fractions (*p* < 0.05 for both fractions, Figure 8b). In the cytosolic fractions, the phosphorylation level of GSK‐3β appears lower in CKO compared to Con although there was no statistical difference. p‐GSK‐3β in CKO was significantly decreased at mitochondrial‐associated membranes (MAMs) (*p* < 0.05, Figure 8c). This indicates higher activity of GSK‐3β at the SR/mito fraction of CKO hearts than in control hearts. Higher GSK‐3β activity could lead to increased interaction of the IP3R‐Grp75 (glucose‐regulated protein 75)‐VDAC (voltage‐dependent anion channel) complex (Paillard et al., 2013) and may explain the lower amount of Ca^2+^ we found in the SR may be due to a subsequent increase in mitochondrial Ca^2+^ uptake.

## 4 DISCUSSION

This study uses Torin1, an mTORC1 and mTORC2 inhibitor, to show that inhibition of mTOR signaling results in decreased functional recovery against I/R injury in *ex vivo* Langendorff‐perfused hearts. In an attempt to replicate this result using a genetic knockout model of mTOR, we observed that interestingly, loss of cardiac mTOR improves cardiac functional recovery in the same model. Despite cardiac mTOR knockout initially seeming to promote post‐I/R recovery, during reperfusion, we observed irregular contractility in our CKO hearts that was not present in Con hearts. When we investigated these hearts on a cellular level, CMs isolated from CKO hearts exhibit weaker contraction and smaller calcium transients at baseline, compared to that of CMs from Con hearts. Caffeine‐induced SR calcium release in isolated CMs suggests that the total of SR calcium content is lower in CKO than that in Con CMs. Western blotting shows that a significant amount of mTOR locates to the SR and mitochondria and decreased phosphorylation of GSK‐3β, a key factor for calcium mobilization in the SR. Those findings suggest that cardiac mTOR located to the SR and mitochondria plays a vital role in CM Ca^2+^ handling, EC coupling and cell survival in I/R injury.

Rapamycin is an mTORC1 inhibitor that inhibits the mTORC1‐S6K1 axis, which suppresses the negative feedback from S6K1 to IRS‐1. The disruption of this negative feedback via rapamycin restores IRS‐1 activity and enhances the cell survival effects of Akt (O'Reilly et al., 2006; Wang et al., 2008). In the heart, treatment with rapamycin‐suppressed apoptosis and preserved cardiac function in *in vivo* “straight MI” models without reperfusion, which was accompanied by an increase in Akt Thr^308^ phosphorylation—a target of PDK1 (Di et al., 2012). In this study, *ex vivo* perfused hearts treated with Torin1, an mTOR inhibitor that selectively binds to the ATP‐binding pocket of mTOR (Liu et al., 2010), suggested that both mTORC1 and mTORC2 activity are required for protecting hearts against I/R injury. The results of this study are consistent with our previous report, in which we demonstrated that overexpression of mTOR was cardioprotective in both *ex vivo* and *in vivo* settings. In that study, we used mTOR‐Tg mice that exhibited mTOR activation in both mTORC1 and mTORC2 pathways and downstream targets (Aoyagi et al., 2012). We also observed that Torin1 suppressed the mTORC2 signaling pathway more dominantly than the mTORC1 signaling pathway. Those findings are consistent with the previous data that showed that Akt activation regulates cell survival in the heart (Matsui et al., 1999, 2001).

Previously, several groups determined the effects of mTOR gene silencing in the hearts using cardiac‐specific mTOR knockout mice with Cre‐loxP system (Mazelin et al., 2016) or cardiac‐specific tamoxifen‐inducible mTOR knockout mice with MerCreMer‐loxP system (Zhang, Contu, et al., 2010). These studies suggest knockout of mTOR leads to dilated cardiomyopathy in both neonatal and adult mice due to increased apoptosis, autophagy, and altered mitochondrial structure. Based on these studies and our Torin1 data, it was therefore surprising when our study using mTOR CKO mice showed that genomic deletion of cardiac mTOR preserved cardiac function and prevented myocardial injury in an *ex vivo* Langendorff I/R model. Unlike other cardiac‐specific mTOR knockout mouse models (Mazelin et al., 2016; Zhang, Contu, et al., 2010), the size and shape of the heart in our mTOR CKO were comparable to the heart from control mice. The level of gene silencing was milder in our mTOR CKO compared to the cardiac‐specific mTOR knockout mice. The mild suppression in the mTOR signaling pathway in both mTORC1 and mTORC2 might cause cardioprotection against I/R injury. Our study went on further to determine a potential cause for the better LVDP recovery after *ex vivo* I/R injury of the CKO hearts. From our data, we hypothesized that alteration in Ca^2+^ handling led to a lower amount of SR Ca^2+^. A decrease in SR Ca^2+^ would also limit the rate of Ca^2+^ influx into the mitochondria and decrease cell death, as Ca^2+^ is a key stimulus for mPTP opening (Crompton et al., 1987; Kung et al., 2011). Opening of the mPTP is a major trigger for inducing programmed necrosis (Kung et al., 2011). Ca^2+^ leak in mTOR CKO hearts would result in less Ca^2+^ entry into the mitochondria, reduced opening of the mPTP, and decreased cell death (Baines, 2010; Garcia‐Dorado et al., 2012; Kung et al., 2011). However, the change in SR Ca^2+^ is not a favorable change with regard to cardiac function, because modulation of Ca^2+^ transients is a key regulatory system in E‐C coupling.

Sarcomere length shortening and time to peak shortening were significantly reduced at baseline in *in vitro* CMs from the CKO hearts compared to Con, whereas there was no difference in baseline systolic function between the Con and CKO hearts in *in vivo* and *ex vivo* experiments. CMs from heart failure subjects typically display decreased Ca^2+^ transients, enhanced diastolic SR Ca^2+^ leak, and diminished SR Ca^2+^ sequestration (Luo & Anderson, 2013). The major mechanisms leading to these phenomena are observed with hyper‐phosphorylation of the ryanodine receptor and reduced SERCA expression and activity (Marx et al., 2000; Mercadier et al., 1990; Meyer et al., 1995; Ono et al., 2000). However, we did not find any evidence that these proteins were changed in CKO hearts (Figure 7). The local Ca^2+^ coupling between the SR and mitochondria is an important contributor for not only the mitochondrial function but also cytosolic Ca^2+^ transient (Garcia‐Perez et al., 2008, 2011; Min et al., 2012). While we observed a lower amount of SR Ca^2+^ in isolated CMs from CKO mice that might count for arrythmia in *ex vivo* hearts, it might not deteriorate cardiac systolic function at baseline in *in vivo* and *ex vivo* hearts. A previous report studying mitochondrial Ca^2+^ transients in CMs suggested that mitochondrial Ca^2+^ uptake *in vivo* is stimulated by additional factors that are not observed in *in vitro* settings (Trollinger et al., 2000). This might explain the discrepancy in CM contraction/cardiac function between *in vitro* and *in vivo*/*ex vivo* settings.

In CMs, the ryanodine receptor and another Ca^2+^ channel, the inositol 1,4,5‐triphosphate receptor 2 (IP3R2), are responsible for the cross‐talk between the SR and mitochondria. IP3R2 contributes to this SR‐mitochondria cross‐talk by tethering to voltage‐dependent anion channel 1 (VDAC1) via the molecular chaperone glucose‐regulated protein 75 (grp75) (Szabadkai et al., 2006). A previous publication suggested mTOR localizes to MAMs and interacts with the IP3R‐Grp75‐VDAC mitochondrial‐tethering complex (Betz et al., 2013). In that study, mTORC2 knockout in mouse embryonic fibroblasts disrupted MAMs, resulting in increased Ca^2+^ uptake and other mitochondrial defects. GSK‐3β was proposed as a potential regulator of the IP3R‐Grp75‐VDAC complex by another report (Gomez et al., 2016). This study proposed a novel role for GSK‐3β by providing evidence that GSK‐3β localizes at MAMs and can physically interact with the IP3R‐Grp75‐VDAC complex and modulate Ca^2+^ transfer between the SR and the mitochondria (Gomez et al., 2016). As mTORC2 is an upstream regulator of Akt and localizes at MAMs, we hypothesized that mTOR knockout might lead to decreased GSK‐3β phosphorylation at serine 9 and serine 21. This decreased GSK‐3β phosphorylation would consequently increase GSK‐3β activity, leading to more Ca^2+^ transfer to the mitochondria and lower amounts of Ca^2+^ in the SR. The increase in GSK‐3β activity in response to mTOR knockout could explain the lower amount of Ca^2+^ in the SR we observed. Previous studies showed that chronic cardiac‐specific mTOR gene silencing exhibited dilated cardiomyopathy (Mazelin et al., 2016; Zhang, Contu, et al., 2010). However, these studies did not examine calcium transients in the SR. Further studies are required to define the role of mTOR in cardiac function and calcium transient following I/R injury.

In summary, we found that pharmacological inhibition of both mTOR complexes via Torin1 inhibits recovery and increases cell death in an *ex vivo* model of I/R injury. In contrast, we also found that cardiac‐specific knockout of mTOR was cardioprotective and decreased cell death in an *ex vivo* model of I/R injury in mTOR knockout mice. From this, we found that SR Ca^2+^ was diminished in CKO hearts, and this change was potentially caused by increased activation of GSK‐3β. This, in turn, leads to additional Ca^2+^ entry into the mitochondria from the SR. Despite the improved post‐I/R recovery in CKO mouse hearts, there was a cost at the cellular level, as CKO mouse CMs showed weaker and more inconsistent contractions post‐I/R. Altered Ca^2+^ regulation is a typical characteristic of heart failure, which may explain the impaired function of individual CKO CMs after reperfusion. An increased understanding of the mechanisms behind post‐I/R dysregulation of Ca^2+^ after can lead to the identification of new mechanistic targets to prevent the progression of heart failure.

## DISCLOSURE

No conflicts of interest, financial, or otherwise, are declared by the authors.

## AUTHOR'S CONTRIBUTIONS

Conception and design of research: B.K.S., N.Y., T.M.; Performed experiments: B.K.S., N.Y., J.K.H., Y.B., M.K., T.A., T.S.; Analyzed data: B.K.S., N.Y., J.K.H., T.A., T.M.; Interpreted results of experiments: B.K.S., T.M.; Prepared figures: B.K.S., T.M.; Drafted manuscript: B.K.S., J.K.H., T.M.; Edited and revised manuscript: B.K.S., N.Y., J.K.H., Y.B., M.K., T.A., T.S., T.M.; Approved final version of manuscript: B.K.S., N.Y., J.K.H., Y.B., M.K., T.A., T.S., T.M.
